# Tumor versus Stromal Cells in Culture—Survival of the Fittest?

**DOI:** 10.1371/journal.pone.0081183

**Published:** 2013-12-02

**Authors:** Krishna M. Talasila, Narve Brekka, Kjersti Mangseth, Daniel Stieber, Lasse Evensen, Gro V. Rosland, Anja Torsvik, Marek Wagner, Simone P. Niclou, Rupavathana Mahesparan, Olav K. Vintermyr, Rolf Bjerkvig, Janice M. Nigro, Hrvoje Miletic

**Affiliations:** 1 Department of Biomedicine, University of Bergen, Bergen, Norway; 2 Department of Pathology, Haukeland University Hospital, Bergen, Norway; 3 NorLux Neuro-Oncology Laboratory, Department of Oncology, Centre de Recherche Public de la Santé (CRP-Santé), Luxembourg, Luxembourg; 4 Department of Neurosurgery, Haukeland University Hospital, Bergen, Norway; University of Virginia Health Science Center, United States of America

## Abstract

Two of the signature genetic events that occur in human gliomas, *EGFR* amplification and *IDH* mutation, are poorly represented in experimental models *in vitro*. *EGFR* amplification, for example, occurs in 40 to 50% of GBM, and yet, *EGFR* amplification is rarely preserved in cell cultures derived from human tumors. To analyze the fate of *EGFR* amplified and *IDH* mutated cells in culture, we followed the development over time of cultures derived from human xenografts in nude rats enriched for tumor cells with *EGFR* amplification and of cultures derived from patient samples with *IDH* mutations, in serum monolayer and spheroid suspension culture, under serum and serum free conditions. We observed under serum monolayer conditions, that nestin positive or nestin and SMA double positive rat stromal cells outgrew *EGFR* amplified tumor cells, while serum spheroid cultures preserved tumor cells with *EGFR* amplification. Serum free suspension culture exhibited a more variable cell composition in that the resultant cell populations were either predominantly nestin/SOX2 co-expressing rat stromal cells or human tumor cells, or a mixture of both. The selection for nestin/SMA positive stromal cells under serum monolayer conditions was also consistently observed in human oligodendrogliomas and oligoastrocytomas with *IDH* mutations. Our results highlight for the first time that serum monolayer conditions can select for stromal cells instead of tumor cells in certain brain tumor subtypes. This result has an important impact on the establishment of new tumor cell cultures from brain tumors and raises the question of the proper conditions for the growth of the tumor cell populations of interest.

## Introduction

Human glioblastoma is a disease that exhibits underlying genetic heterogeneity even within individual tumors. Amplification of specific receptor tyrosine kinases is one visible measure of the genetic diversity of cells within a tumor, but the biological properties of these cells remain ill defined, in part, because few *in vitro* models retain these cells. *EGFR* amplification, for example, is a signature genetic event for human glioblastoma, and yet few cell populations derived from human glioblastoma biopsies ever maintain *EGFR* amplification in culture [1], [2]. In contrast, *EGFR* amplification appears to be selected for in certain xenograft systems when generated directly from primary tumors [2], [3], [4]. While *EGFR* amplification may represent a small percentage of cells in the primary tumor, engraftment into rodents intracranially appears to select specifically for these cells for reasons that are currently not well understood [4]. However, once placed into culture, amplifications “disappear,” even from xenograft material where the proportion of *EGFR* amplified cells is in great excess of the cells that do not harbor amplification units [2], [3].

A second tumor genotype that has been difficult to establish in experimental systems are gliomas with *IDH* mutations, in particular oligodendrogliomas. Attempts to passage them in culture have led to only a few successes, and some additional cell cultures appear to lose the cells harboring *IDH* mutation [5], [6], [7], [8], [9]. These results indicate that the genotype of some tumor cells is not always compatible with culture conditions despite their obvious deleterious effects within the human brain. However, rigorous genetic and phenotypic analysis of the cells that do grow has not been performed in all studies [5].

There are many possibilities for the apparent disappearance or absence of these genotypes in culture. In addition, to understand why we might lose these genotypes in culture could broaden our understanding of essential parameters for the growth of these tumors in the human brain.

In this work, we discovered that a progenitor-like cell population outgrows specifically *EGFR* amplified tumor cells in serum free conditions and that under adherent serum conditions, a nestin/SMA positive cell population consistently emerges from human *EGFR* amplified xenografts and *IDH* mutated primary human biopsies. Our results indicate that these culture conditions favor the growth of normal cell types at the expense of certain tumor cells *in vitro*.

## Materials and Methods

### Ethics statement

Patient material was obtained from surgeries performed at the Haukeland University Hospital (Bergen, Norway). Written consent was obtained from patients with procedures that were approved for the project (project number 013.09) by the Regional Ethics Committee (Bergen, Norway). All animal protocols were approved by authorities in an AAALAC accredited facility at the Haukeland University Hospital and in accordance with the national regulations of Norway.

### Dissociation of tumor tissue

Tumor tissue (<1 g) was gently cut with scalpel blades, rinsed in Hank's buffered saline (HBSS), and enzymatically dissociated at 37°C for one hour, or until single cells could be observed, in 5 ml of HBSS containing Liberase DH (0.25 mg in 5 ml; Roche, Basel, Switzerland) and 100  DNAse (2% solution in HBSS; Sigma, St Louis, MO). Dulbbeco's modified eagle medium (DMEM) with 10% serum was added, and the enzyme mixture was removed after centrifugation. The sample was resuspended in DMEM with 10% serum, gently triturated with a 10 ml plastic pipet, and filtered through a 70  filter. Blood cells were subsequently lysed with EasyLyse (DAKO, Glostrup, Denmark), and tumor cells were washed thoroughly two times with 20 ml of Dulbecco's phosphate buffered saline without calcium and magnesium.

### Cell culture

Biopsy spheroids from GBM xenografts were prepared and cultured as described previously [10]. For cultures in Neurobasal Medium (NBM), tumor cells were resuspended in NBM supplemented with B27 supplement, Glutamax (NBM; Life Technologies, Carlsbad, CA), FGF2 (20 ng/ml), and EGF (20 ng/ml; Peprotech, Rocky Hill, NJ) over non-treated culture flasks (Nunc, Penfield, NY). Serum monolayer cell cultures were maintained in DMEM supplemented with 10% fetal calf serum (FCS) and 1% glutamine. Medium was changed once/twice weekly. Cells were grown at 37°C in a humidified atmosphere of 5% CO_2_. For osteogenic and adipogenic differentiation, monolayer cultures were incubated in appropriate medium for 21 days (StemPro Differentiation Kits, Life Technologies), fixed in 4% paraformaldehyde, and stained with alizarin red or oil red to detect for bone and fat, respectively.

### Capillary network formation assay

The *in vitro* human umbilical cord vein endothelial cell-pulmonary artery smooth muscle cell (HUVEC-PaSMC) co-culture angiogenesis assay has been described previously [11]. Briefly, HUVEC (GFP-transduced for visualization) and PaSMC were counted and co-seeded in a 96-well-plate and centrifuged briefly at 200×g to achieve even distribution of cells in the wells. Co-cultures were incubated under standard cell culture conditions in EGM-2 medium for 72 hours to allow network formation. Cell numbers and culture volume were as follows (per well): PaSMC, 5×10^4^, HUVEC 10×10^3^, 200 EGM-2. The cells from low grade glioma monolayer cultures replaced either HUVEC or PaSMC in the assay. For HUVEC replacement, visualization of the test cell populations was attempted with an endothelial cell marker, lectin, which was conjugated to FITC.

### DNA isolation

DNA was isolated from patient material that had been immediately frozen in liquid nitrogen subsequent to surgery. Frozen sections were prepared, and every fifteenth section was stained with H&E to ensure >60% tumor cell content. Samples were treated with proteinase K overnight at 50°C in ATL buffer (Qiagen) and then DNAse free RNAseA (Thermo Fisher Scientific, Waltham, MA) for 5 min at room temperature. Reactions were extracted with phenol:chloroform:isoamyl alcohol 25∶24∶1 saturated with 10 mM Tris, pH 8.0, 1 mM EDTA (Sigma) and precipitated in 2.5 M NH_4_OAc and 2.5 volumes of 100% ethanol. DNA was resuspended in Nuclease-Free water (Qiagen).

### Paraffin embedding of cultured cells

Spheroids/cell pellets were washed with PBS and fixed in 4% formalin overnight at room temperature. Cells were centrifuged for 10 min. at 300×g, and formalin was discarded. The cell pellet was then stained with 20  of 0.5% Methyl green solution (Sigma) for 2 min. The stained pellet was washed in 5 ml of PBS and centrifuged for 10 min. at 300×g. The pellet was mixed properly first with 20  of human plasma (from volunteer) and then with 10  of human Thrombin (100 U/mL, Merck KGaA, Darmstadt, Germany). The stained pellet was finally processed for paraffin embedding

### CGH+SNP arrays and data analysis

DNA was digested using the restriction enzymes RSA1 and Alu1 and labeled using the BioPrime aCGH Genomic Labeling Kit (life technologies) and Cy3 and Cy5 dyes (GE Healthcare, Buckinghamshire, UK)), following standard protocols for Agilent CGH+SNP. A female HapMap sample with a known genotype (European female, NA12878_V1) provided by the Coriell Repository (Camden, NJ) was used as a reference for each of the CGH+SNP experiments. Labeled DNA was competitively hybridized to SurePrint G3 Human 2×400 k CGH+SNP microarrays (G4842A, Agilent Technologies, Santa Clara, CA) following standard Agilent protocols. The slides were scanned at 3  resolution using the Agilent High-Resolution Microarray scanner and the image data were extracted using Feature Extraction (Agilent Technologies). Feature extraction files were imported into Genomic Workbench 7.0 (Agilent Technologies) for visualization and analysis. For CGH, after diploid centralization and GC correction, aberrations were called using the ADM2 algorithm with a threshold setting of 20, centralization on with threshold of 25 and an aberration filter min Probes = 5 and minAvgAbsLogRatio = 0.35 for amplifications and deletions.

### DNA fingerprinting

Oligodendroglioma cell populations used for implantation or culture experiments were fingerprinted to confirm that all cell populations were derived from the matched patient biopsy. The AmpFlSTR Profiler Plus PCR Amplification Kit (life technologies) was used according to the manufacturer's protocol. This kit amplifies nine tetranucleotide short tandem repeat (STR) loci and the amelogenin (gender determination) locus in a single reaction. The samples were run and allele sizes interpreted on an ABI3100 Genetic Analyzer (life technologies). The fingerprinting profiles were run through the online international reference STR profile database for human cell lines at the German collection of microorganisms and cell cultures DSMZ http://www.dsmz.de. Profiles were analyzed to determine that they matched the original biopsy, and that they did not match any of the cell lines listed in the DSMZ STR database.

### Immunohistochemistry

Immunohistochemistry of paraffin sections was performed as described previously [12]. Freshly cultured cells were fixed in acetone for 10 min. at −20°C, blocked against endogenous hydrogen peroxidase in 0.3% hydrogen peroxide for 25 min, blocked with 5% serum for 1 hour at room temperature, and incubated with primary antibody for 1 hour at room temperature. The Vectastain ABC kit was used for detection of the primary antibody. The following primary antibodies were used: anti-human nestin (1∶200; Millipore, Billerica, MA), anti-rat nestin diluted 1∶200 (Millipore), anti-human SOX2 (1∶200; R&D Systems, Minneapolis, MN), anti-EGFR (1∶500; Santa Cruz, Dallas, TX), anti-GFAP diluted 1∶500 (Millipore), anti-beta-tubulin III (1∶100; Millipore), anti-SMA (1∶200; Abcam, Cambridge, UK), anti-vWF (1∶500; DAKO), and anti-IDH1 R132H (1∶80; Dianova, Hamburg, Germany). The H&E and immunohistochemical stainings were analyzed on a Zeiss light microscope (Zeiss, Jena, Germany) using Zeiss imaging software. Overview pictures of histological slides were taken by using a digital slide scanner and Imagescope software (Aperio, Vista, CA).

### FISH

FISH analyses of paraffin sections were performed with the Vysis LSI EGFR SpectrumOrange/CEP 7 SpectrumGreen probe (Abbott Molecular, Des Plaines, IL) using the DAKO Histology FISH Accessory Kit (DAKO).

### *In Vivo* experiments

Nude immunodeficient rats (rnu/rnu Rowett) were fed a standard pellet diet and were provided with water ad libitum. Biopsy spheroids or cell supensions were stereotactically implanted into the right brain hemisphere as described previously [13]. Cell suspensions from oligodendroglioma monolayer cultures were implanted into 6 to 8 week old NOD/SCID mice of either gender (Klink et al). Coordinates for implantation were 1.5 mm to the right and 0.5 mm behind the bregma suture. Maximum penetration of the syringe was 2.0 mm into the tissue with injection of the cells at 1.5 mm. Rats or mice were euthanized with CO_2_, perfused intracardially with 0.9% NaCl, and sacrificed when symptoms developed

### Magnetic resonance imaging

Axial (T2w) RARE sequences were acquired as described previously [14].

## Results

### *EGFR*-amplified glioblastoma cells are enriched in a xenograft model based on biopsy spheroids

**Figure 1:**
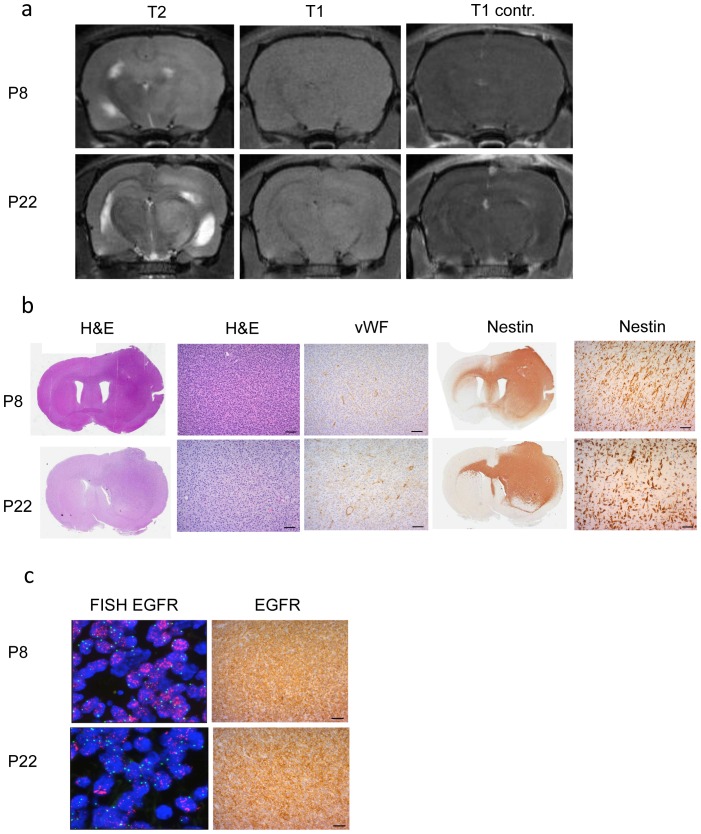
Characterization of EGFR-amplified, invasive human glioblastoma xenografts. (**a)** T2- and T1-weighted MRIs with and without contrast show invasive lesions with ill-defined borders and no contrast enhancement in two EGFR amplified xenograft tumors (P8 and P22). (**b**) Hematoxylin and eosin (H&E) and immunohistochemical staining of sections with antibodies against vWF and human-specific nestin. Higher magnification shows invasive tumor areas. **(c)** FISH with an *EGFR*/Chromosome 7 probe in red and green, respectively, and immunohistochemical staining of sections with antibodies against EGFR. Scale bars 50 .

We cultured biopsy spheroids derived from three *EGFR* amplified patient biopsies (Table S1) and passaged them over several generations in the brains of nude rats as described previously [4]. Highly invasive tumors developed even after multiple passages with no contrast enhancement on MRI (Figure 1a). Histology confirmed the highly invasive nature of the xenografts with single cells infiltrating into normal brain tissue (Figure 1b). FISH analysis and immunohistochemistry for EGFR showed high *EGFR* amplification and strong expression in the tumor cells, respectively (Figure 1c), indicating that the xenograft model selected for highly EGFR amplified/expressing cells as described previously [4].

### *EGFR* amplification and expression is preserved only under serum spheroid conditions

**Figure 2:**
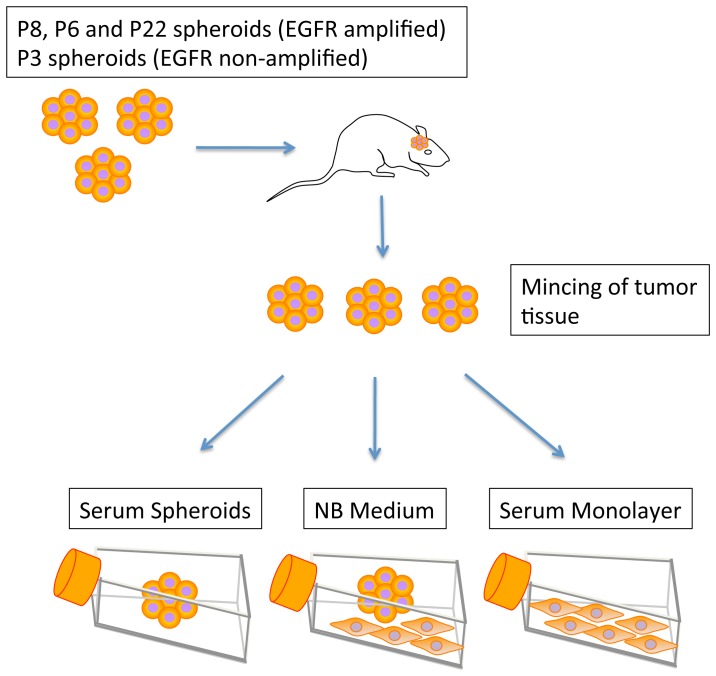
GBM cells derived from xenografts are cultured under three different conditions. Biopsy spheroids from three EGFR-amplified GBM and one EGFR non-amplified GBM (control) were implanted into the brain of nude rats. Established tumors were minced and cultured under three different conditions. 1. Spheroid serum culture. 2. Monolayer serum culture. 3. Neural Basal Medium (NBM) serum free cultures

**Figure 3:**
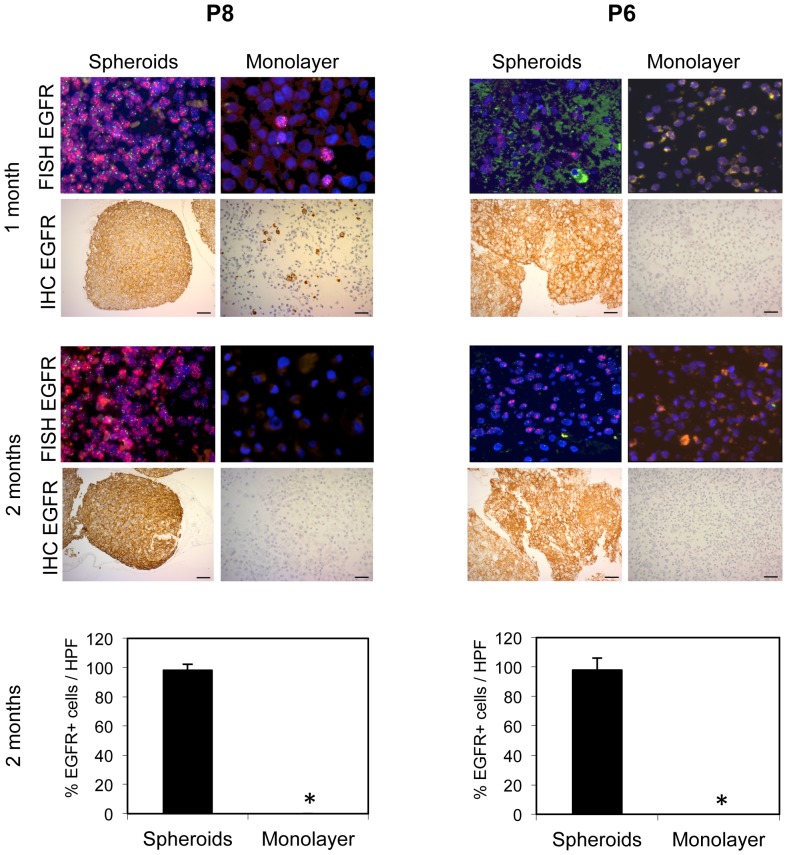
EGFR amplification and expression is preserved in serum spheroid cultures and “lost” in monolayer cultures. Cultured spheroids or single cells were coagulated, fixed with 4% formalin, and embedded in paraffin. FISH with an *EGFR*/Chromosome 7 probe in red and green, respectively, and immunohistochemical staining with antibodies against EGFR. In monolayer culture, both EGFR amplification and the chromosome 7 probe are not detectable after two months in cultures from both xenografts. Immunhistochemical staining for EGFR is also negative. Scale bars 50 µm. Quantification of EGFR expressing cells in three random high power (400×) microscopic view fields (HPF) in each group. Asterix indicates 0%. Values represent mean ± s.d.

From the passaged xenografts, tumor tissue was introduced into culture under three different conditions: serum monolayer, and spheroid suspension cultures in serum or Neural Basal medium (NBM; Figure 2). Serum monolayer cultures have been the traditional method for the development of glioblastoma cell lines *in vitro* until it was discovered that spheroid suspension cultures in NBM or serum more faithfully preserve the genotype of the patient tumor relative to serum monolayer cultures [13], [15]. We first investigated the difference between serum monolayer and serum spheroid cultures where the culture media are identical. After 1, 2, and 3 months in culture, we analyzed the tumor cells under the different conditions for the presence of EGFR amplification and expression, by FISH and immunohistochemistry, respectively. Whereas EGFR amplification and expression was preserved in spheroid suspension cultures in serum, it had disappeared or was highly reduced after 1–3 months in serum monolayer cultures derived from all xenografts (Figure 3; Figure S1a). Quantification of EGFR+ cells confirmed these differences (Figure 3; Figure S2).

The unexpected result from the FISH experiment, where probes for EGFR and chromosome 7 (chromosomal location of EGFR) were used, was that cells without EGFR amplification also appeared to have “lost” human chromosome 7, as the chromosome 7 FISH probe (green) also did not hybridize to the cells in serum monolayer cultures. The selection process to the human chromosome 7 deficient cell population occurred rapidly in cultures from P8 and P6, where most of the EGFR amplified cells had already disappeared after one month (Figure 3). In P22, only after three months, did the majority of cells seem to lose chromosome 7 (Figure S1a). In addition, there was never a complete conversion of the cultures to the chromosome 7 deficient cells in the P22 cultures, as FISH revealed single cells with *EGFR* amplification. These cells also expressed EGFR, although at a reduced level compared to spheroid cultures, as verified by immunohistochemistry (Figure S1a and S2).

In effect, tumor cells with *EGFR* amplification no longer remained, or were dramatically reduced in serum monolayer. In contrast, serum spheroid cultures preserved *EGFR* amplified cells, as described previously [4].

### Selection of glioblastoma versus stromal cells is dependent on the culture conditions

**Figure 4:**
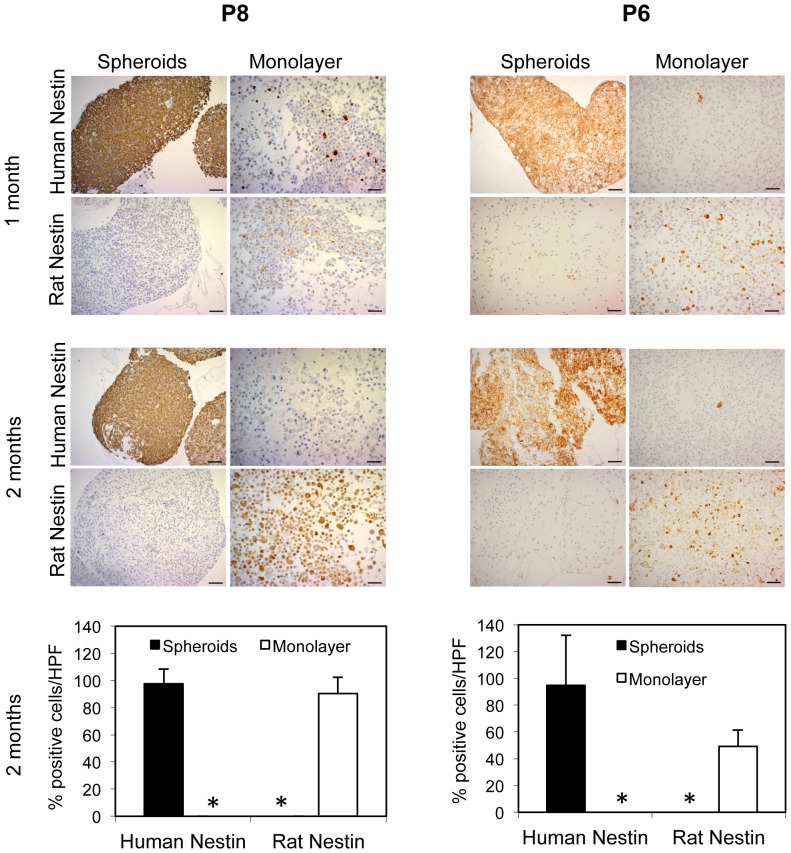
Rat stromal cells have a selective growth advantage over EGFR amplified cells in serum monolayer culture. Cultured spheroids or single cells were coagulated, fixed with 4% formalin, and embedded in paraffin. Immunohistochemical staining was performed with antibodies against human-specific and rat-specific nestin. After two months, nestin-expressing rat cells take over at expense of human tumor cells in serum monolayer culture. As expected, nestin-expressing human cells are predominant in serum spheroid cultures. Scale bars 50 µm. Quantification of human and rat nestin expressing cells in three random high power (400×) microscopic view fields (HPF) in each group. Asterix indicates 0%. Values represent mean ± s.d.

One possibility for this result was that the cells taking over the serum monolayer cultures were no longer human, but instead from the rat, and could represent a stromal cell population that has a growth advantage *in vitro*. To verify that rat stromal cells outgrew *EGFR* amplified tumor cells from the three xenografts under different *in vitro* conditions, we used human- and rat-specific anti-nestin antibodies to detect and differentiate between cells from the different species. Human nestin was used as a marker for the presence of human tumor cells, as it is highly expressed in the tumor cells of our xenograft model [4], [13]. In addition, we were also able to use a rat-specific ant-nestin antibody, to follow development of the outgrowth of stromal cell populations under serum monolayer conditions. Nestin is a marker often associated with neural progenitor and glial cells, which have been shown to comprise stromal cell populations within brain tumors [16]. Under serum spheroid conditions, the majority of cells from all xenografts was positive for human nestin as would be expected from a human glioma cell population. In contrast, under serum monolayer conditions, a fraction of rat-nestin positive cells that were initially present eventually predominated over time in cultures derived from all xenografts (Figure 4; Figures S1b and S2).

### NBM cultures select for stromal cells or tumor cells with preserved EGFR amplification, but strongly reduced EGFR expression

**Figure 5:**
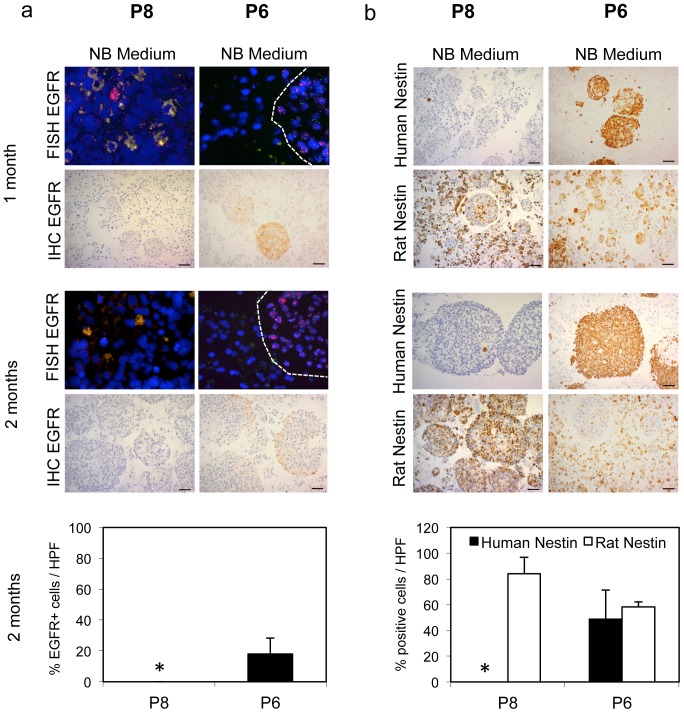
Rat stromal cells or a combination of tumor and stromal cells have a growth advantage in serum free cultures. **(a)** FISH with an *EGFR*/Chromosome 7 probe in red and green, respectively, and immunohistochemical staining with antibodies against EGFR. In P8 cultures, both EGFR amplification and the chromosome 7 probe are not detectable after two months. In P6 cultures, EGFR amplification is preserved in spheroids (dotted line), while both EGFR amplification and the chromosome 7 probe are not detectable in single cells. EGFR expression is lost in P8 cultures, while it is still detectable at a low expression level in spheroids from P6 cultures. Quantification of EGFR expressing cells in three random high power (400×) microscopic view fields (HPF) in each group. Asterix indicates 0%. Values represent mean ± s.d. **(b)** Immunohistochemical staining with antibodies against human-specific and rat-specific nestin. In P8 cultures, rat-nestin positive stromal cells have a growth advantage over human cells. In P6 cultures, a mix of human and rat cells survive. Human cells form spheroids, while single cells are rat cells. Scale bars 50 µm. Quantification of rat and human nestin expressing cells in three random high power (400×) microscopic view fields (HPF) in each group. Asterix indicates 0%. Values represent mean ± s.d.

Although NBM culture, similar to serum spheroid culture, has been shown to preserve the genotype and phenotype of primary human gliomas, reports about the maintenance of *EGFR* amplification in derived cultures are contradictory [1], [15], [17]. While some investigators observed that *EGFR* amplified cells are not preserved in standard NBM culture, others described the opposite [1], [17]. In fact, when we cultured our three *EGFR* amplified xenografts in NBM, three different scenarios emerged. From P8 tumors, similar to serum monolayer cultures, a cell population was generated that appeared to be derived from the rat, as the chromosome 7 probe had also disappeared along with amplification and expression of EGFR (Figure 5a). However, in P22 NBM cultures, a tumor cell population developed that showed EGFR amplification but strongly reduced EGFR expression after two months in culture (Figure S3a).

Interestingly, P6 revealed a third scenario, cultures where a mix of human tumor and rat stromal cells co-existed. While the human cells mostly formed spheroids, the rat cells remained as single cells (Figure 5a). Similar to P22, P6 tumor cells in NBM still retained *EGFR* amplification, but the expression of EGFR was markedly reduced.

The differences in the cell populations that developed were further confirmed by immunostaining for human or rat nestin and quantified (Figure 5b; Figure S3b).

To test whether stromal cells could be derived from a GBM of a different genotype, we performed the same cell culture experiment with a human GBM xenograft (P3) without EGFR amplification (Figure S4; Table S1). Under all three different conditions, human tumor cells predominated in the cultures from P3 (Figures S5 and S6).

### Confirmation of the stromal/tumor cell populations by *in vivo* experiments

Serum spheroid cultures derived from the *EGFR* amplified glioblastoma xenograft P8 when implanted into the brain of nude rats resulted in highly invasive tumors as described previously [4]. In contrast, the cells from NBM and serum monolayer cultures did not form tumors as verified by MRI and histology (Figure S7).

### Stromal rat cells represent nestin+ SOX2+ neural progenitor cells or nestin+ SMA+ cells

**Figure 6:**
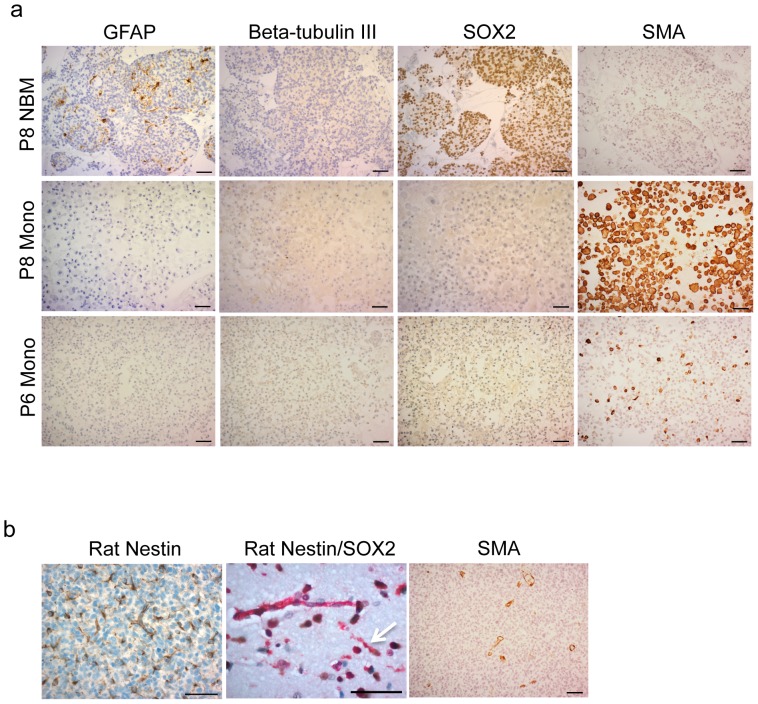
Characterization of rat stromal cells *in vitro* and *in vivo*. **(a)** Rat stromal cells from P8 NBM, and P8 and P6 monolayer cultures were stained with antibodies against GFAP, beta-tubulin III, SOX2 and SMA. Rat cells from P8 NBM cultures are positive for SOX2 and negative for SMA, while monolayer cells are negative for SOX2 and positive for SMA. **(b)** Xenografts were stained with antibodies against rat nestin, SOX2 and SMA. Rat nestin is positive on tumor vessels and single cells in human *EGFR* amplified GBM xenografts *in vivo*. Double staining for rat nestin and SOX2 shows rat nestin+/SOX- vessels and rat nestin+/SOX2+ single cells (arrow). SMA is positive on vessels. Scale bars 50 µm.

In order to characterize the stromal cell populations from *EGFR* amplified xenograft cultures, we performed immunostaining on cells from serum monolayer cultures derived from P8, P6, and P22 as well as NBM cultures derived from P8 with SOX2, GFAP, beta-tubulin-III, and SMA. Stromal cells in P8 NBM cultures were positive for SOX2 and negative for GFAP and beta-tubulin III, indicating that these cells are most likely nestin+ SOX2+ neural progenitors (Figure 6a). The stromal cells in monolayer cultures were negative for SOX2, GFAP, and beta-tubulin III, but they were positive for SMA. The SMA positivity was variable as the majority of stromal cells in P8 were positive for SMA, while in P6 cultures only a fraction of these cells was positive (Figure 6a). In P22 cultures, 52  7% of cells were positive for SMA, while the GFAP (27  5%), beta-tubulin III (21  9%), and SOX2 (36  3%) positive cells are most likely human tumor cells as there was not a complete selection towards rat stromal cells (Figure S8).

In order to determine whether the cultured stromal cells correlated with specific cell types *in vivo*, staining for nestin, SOX2, and SMA was performed on paraffin sections from the GBM xenografts. Vessels as well as single cells within P8 and P22 xenografts were positive for rat nestin (Figure 6b). SOX2 and SMA distinguished two nestin staining cell populations as SOX2 was localized to the individual stromal cells within tumors and not vessels, whereas SMA stained only the vessels (Figure 6b). Thus, nestin+ SOX2+ as well as nestin+ SMA+ double positive cells could be identified *in vivo* in the xenograft models.

### Derivation of a stromal cell population from human low grade gliomas

**Figure 7:**
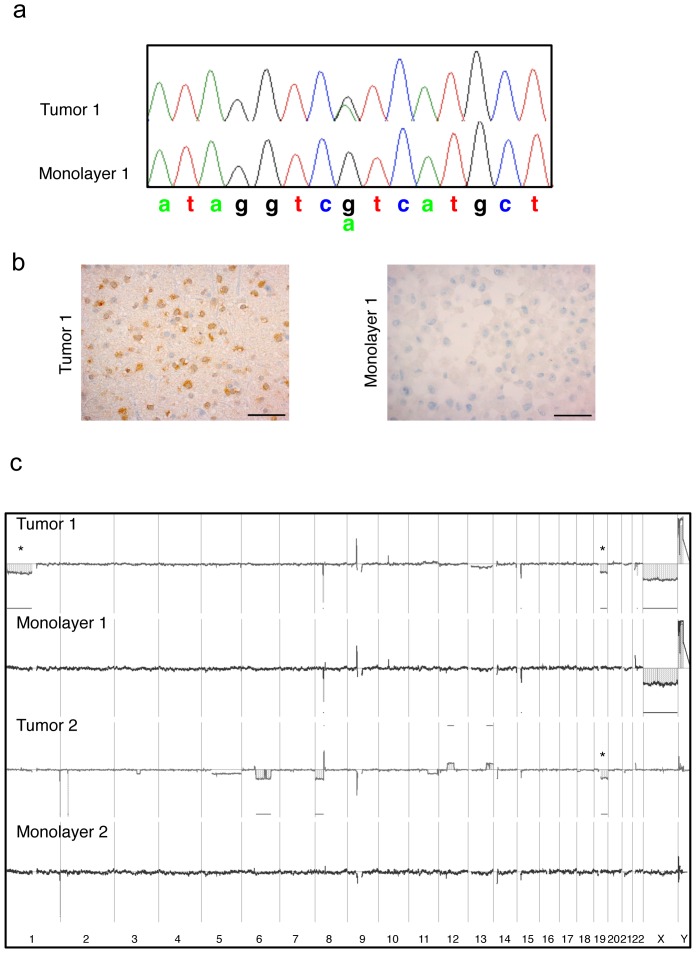
Genomic profiles are normal in monolayer cell populations derived from human oligodendrogliomas. **(a)** Sanger sequencing demonstrates that the *IDH1* mutation of the primary tumor is not preserved in the derived monolayer cell population. **(b)** Immunostaining with antibodies against IDH1 R132H confirms that monolayer cells are negative and the primary biopsy strongly positive. **(c)** aCGH profiles for the derived cell populations and the matching human oligodendroglioma are shown. Data is shown in log_2_ space across the genome where log_2_ 0 (at the midline) indicates the normal diploid copy number. Asterisks (*) highlight 1p and 19q losses that are typical of oligodendroglioma.

That stromal cell populations preferentially grow in serum monolayer, was an observation that we also made in our attempts to derive tumor cell populations from primary human oligodendroglioma and oligoastrocytoma biopsies that frequently harbor *IDH* mutations. Dissociated cells from primary tumors were initially introduced into the same three culture conditions as described above for EGFR amplified cells. Expanding cell populations were derived only from serum monolayer culture, whereas the spheres that developed in serum suspension culture appeared to be a layer of non-tumor cells surrounding a core made up of extracellular matrix components (data not shown). Serum free cultures yielded viable spheres that did not proliferate *in vitro* but in one case, engrafted [7]. In contrast, within two weeks, an adherent, highly proliferative cell type grew out of the serum monolayer cultures from primary biopsies of four oligodendroglioma and one oligoastrocytoma. In order to confirm whether these cell populations were derived from tumor or normal cells, two types of genetic tests were performed. In the first test, we analyzed cell populations for the *IDH1/2* mutation of the primary tumor. Three out of four oligodendrogliomas and one oligoastrocytoma were all mutated at codon 132 of *IDH1* whereas one oligodendroglioma did not harbor mutations at the canonical codons 132 or 172 of *IDH1* or *IDH2* [18]. Four of these cell populations were tested for the parental tumor *IDH* mutation. In none of these cases did we observe the cells in monolayer to harbor *IDH* mutations (Figure 7a; Table S2). The results were confirmed by immunohistochemistry with an antibody specific for IDH1 R132H mutation (Figure 7b). In the second test, aCGH was performed on DNA isolated from patient tumors, including the one case without *IDH* mutation and the derived monolayer culture (Figure 7c; Table S2). In two of the cases tested, we observed a pattern of aCGH indicative of a normal genotype whereas the tumor profiles showed gross chromosomal changes typical of human oligodendrogliomas, such as 1p and 19q losses (Figure 7c). Fingerprinting of the adherent cell populations was subsequently performed to confirm that they were indeed derived from the matched primary human biopsy (data not shown).

Cells from monolayer cultures of a human oligodendroglioma were implanted into the brains of NOD/SCID mice, but were not tumorigenic (Figure S9).

### Characterization of stromal cells derived from human low grade glioma

**Figure 8:**
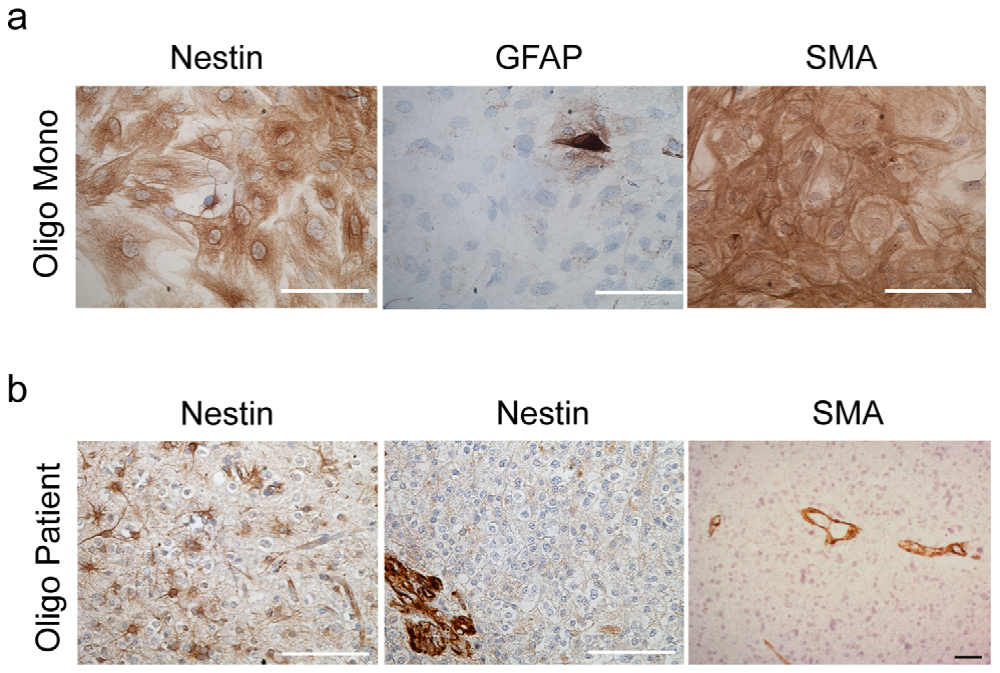
Characterization of stromal cells derived from human oligodendroglioma *in vitro* and *in vivo*. **(a)** Human stromal cells from oligodendrogliomas, cultured under monolayer conditions are positive for nestin and SMA. Only few single cells are positive for GFAP. **(b)** Nestin is localized to reactive astrocytes and the vasculature in human oligodendroglioma samples *in vivo*. SMA is positive on the vasculature. Scale bars 50 µm.

The human cells derived from primary oligodendroglioma had the morphology of so-called mesenchymal stem cells (Figure 8a) that are often derived from primary tumor biopsies of various tissue types [19], [20]. These cells were also characterized by immunostaining with nestin and SMA, and GFAP as well to identify astrocytes. The majority of cells in every cell population generated was highly positive for nestin, whereas few cells were positive for GFAP (Figure 8a). In addition, just as for the rat stromal cells in the GBM xenograft model, the human monolayer cultures were positive for SMA (Figure 8a).

In primary biopsies from lower grade gliomas, the vasculature was always nestin and SMA positive and some nestin positive reactive astrocytes were present in WHO grade II cases, whereas the actual tumor cells were variably positive (Figure 8b). Previous studies have similarly observed nestin staining of the vasculature in primary low grade glioma samples [21], [22], [23]. In an oligodendroglioma xenograft, host nestin positive cells were found in the subependymal areas where neural stem cells reside, but the vasculature was also positive regardless of proximity to the tumor [7].

### Adherent human cells from human oligodendrogliomas fail to promote capillary-like networks

Based on the *in vivo* and *in vitro* immunostaining, the adherent cultures whether derived from the xenografts or human biopsies are likely to be the same cell population and perhaps functionally related to the vasculature. To test whether the adherent cells might be a cell type derived from the vasculature, a HUVEC-PaSMC co-culture capillary network formation assay was employed [11]. In this assay, in contrast to Matrigel tube formation assays, only endothelial cells are able to form capillary-like networks driven by the VEGF-secreting and extracellular matrix-depositing PaSMC. Monolayer cell populations isolated from two different oligodendrogliomas, in separate co-cultures, replaced either HUVECs or PaSMC, to determine whether they could form capillary-like networks or drive HUVEC network formation, respectively. Because the derived cell populations did not bind the FITC-labeled lectin, an endothelial cell marker, results were inconclusive for their ability to form capillary networks when used as the putative endothelial cell in the assay (data not shown). However, the cell populations were clearly unable to drive HUVEC network formation when replacing the PaSMC in the assay (Figure S10a). These results indicate that these derived cell populations are either not functionally related to the vasculature or have lost these attributes in serum monolayer culture.

### Differentiation of adherent cell populations into bone

One additional feature of all of the derived adherent cell populations is that they have a mesenchymal stem cell-like phenotype in terms of morphology, nestin/SMA staining, and growth in adherent culture in serum [24]. Mesenchymal stem cell-like stromal cells have been derived from human GBM xenografts as well as from primary human tumor types *in vitro,* and experiments indicate that these cells may originate from the vasculature [19], [20], [25], [26]. Isolated mesenchymal stem cell-like stromal cells also have the ability to differentiate into bone, cartilage, and fat as mesenchymal stem cells [24]. To further characterize the adherent cell populations, three derived from human oligodendrogliomas were tested for their ability to differentiate into bone and two into fat. All three cell populations differentiated into bone in osteogenic medium, whereas neither of two cell populations tested differentiated into fat in adipogenic medium (Figure S10b). Based on the ability to differentiate into bone but not fat, the derived cell populations are reminiscent of either lineage restricted mesenchymal stem cells or interestingly, similar to endothelial cell populations isolated from a transgenic mouse model of prostate cancer [27].

## Discussion

The results of this work indicate that *in vitro* conditions surprisingly favor the outgrowth of certain non-tumor cell populations even in the case of aggressive human malignant gliomas. Two interesting ideas emerge from this work. The first is that with certain glioma cell types, a normal cell population derived from tumor biopsies often thrives in adherent cell culture at the expense of the tumor cell populations. In the case of low grade gliomas with *IDH* mutations, it remains unclear as to whether tumor cells ever attach. In our experience, we found that an *IDH* mutated anaplastic oligodendroglioma never attached to plastic or even to specific molecules that make up extracellular matrix [7]. In the case of *EGFR* amplified cells from human GBMs, these cells do attach to plastic, as we have shown here, but they seem to not multiply efficiently under *in vitro* conditions. Other investigators have reported that nearly 50% of adherent cell populations from GBM exhibited normal aCGH profiles [28]. The second idea then is why are these tumor cell populations at a disadvantage *in vitro* and can this information ultimately be used for therapy. Our results also emphasize the critical need to understand the specific growth conditions of different cell populations within human tumors.

Adherent serum cultures have been the standard growth conditions for glioma cells since the 1970s. Although it has been shown that this culturing procedure, in contrast to serum-free cultures, does not faithfully preserve the patient genotype [15], cell lines that have been established a long time ago, are still broadly used today, as for example U87 and U251. Interestingly, studies in the 1970s and 1980s reported about the establishment of adherent serum cultures from patient GBM; however, some of these cell lines were non-tumorigenic when implanted into immunodeficient mice [29], [30], [31]. One may speculate whether these were stromal cells similar to the populations described here or tumor cells that have lost the ability to initiate tumor growth *in vivo*. One GBM cell line, SKMG-3, in which *EGFR* amplification has been preserved, was actually established under serum monolayer conditions. However, this cell line has only been used *in vitro* [32], [33], [34] and our attempts to establish tumors from that cell line *in vivo* failed (unpublished observations). As this cell line harbors additional mutations that are typical for GBM, the cultured cells seem to be tumor cells; however, it is still unclear why they are non-tumorigenic [32]. All other attempts to establish *EGFR* amplified glioma cells in adherent serum conditions have failed and led to the assumption that *EGFR* amplification is “lost” during the culturing process [2]. However, our results demonstrate that *EGFR* amplified tumor cells can be easily outgrown by another cell population, which in case of our xenograft model was a stromal cell population. Thus, the proposed “loss” of *EGFR* amplification in monolayer cultures by Pandita et al. [2] is simply a loss of *EGFR* amplified tumor cells at the expense of other cells, which in the case of human biopsies might also be other tumor cells that do not harbor EGFR amplification. A more recent study by Fael Al-Mayhani et al. showed that EGFR amplification is also lost in serum-free monolayer cultures on a defined extracellular matrix, while other chromosomal aberrations are preserved, indicating that EGFR non-amplified tumor cells had a selective growth advantage even in these cultures [35]. A similar study by Pollard et al. describes the efficient expansion of glioma stem cell lines under adherent culture conditions; however, only two cell lines were characterized genetically which did not harbor EGFR amplification [36]. In contrast, serum spheroid cultures seem to be the best conditions to preserve tumor cells with *EGFR* amplification as also observed previously [4]. A recent publication pointed out that NBM cultures can also preserve these cells if EGF is not included or added at very low concentrations [1]. Other investigators have demonstrated that GBM cell populations can be derived in serum-free cultures without any exogenously added mitogenic factors [37]. These studies and our work highlight the need for the definition of optimal culture conditions within the glioma field, which might differ dependent on the genotype of the patient. However, a general conclusion is that adherent serum cultures should, if possible, be avoided as the tumor cells might either not faithfully reflect the genotype of the patient [15] or as in our experiments, be replaced by a stromal cell population.

We cannot accurately identify the *in vivo* precursor/counterpart of the stromal cells that we find in monolayer *in vitro*, but they resemble to some degree mesenchymal stromal cell populations derived from glioma model systems and other solid tumor types [19], [20], [26], [38], [39]. These cell populations have been proposed to be of perivascular origin, such as pericytes, and in some cases, they have been shown to support vascular tube formation of primary endothelial cells [19], [25]. The adherent cell populations derived in our study, however, seem to be limited in mesenchymal stem cell properties, and resemble instead endothelial cell populations isolated from a transgenic mouse model for prostate cancer [27]. However, the cells in our study are deficient in forming/promoting capillary networks, but this result is perhaps due to inappropriate culture in serum as opposed to medium formulated for endothelial cells. The fact that they differentiate into bone is reminiscent of a common histological feature of human oligodendrogliomas, calcifications, the origin of which is currently unknown [40]. Furthermore, calcifications in human oligodendrogliomas have been associated with vessels although not exclusively [41]. The fact that some monolayer cell populations differentiated into bone indicates that a putative source of some calcifications found in human oligodendrogliomas may be derived from a normal cell population that infiltrates the tumors and is perhaps influenced directly by secreted proteins from the tumor. The implication of these results is that calcifications in human oligodendrogliomas might be the result of an active process, such as differentiation of stromal cells that might be influenced by the tumor microenvironment. Other investigators have made similar observations and conclusions regarding calcifications that are often observed in ovarian carcinomas [20].

One additional hypothesis is that the adherent cells are derived from a neural stem/progenitor cell type that becomes differentiated and grows efficiently *in vitro* as opposed to *in vivo*. In serum monolayer, for example, the cells may lose SOX2 and in so doing, become mesenchymal stem cell-like. Interestingly, when assessing the differentiation potential of cortical stem cells *in vitro*, investigators found that plating density affected cellular fate. Lower plating density of the stem cells generated a cell population that was SMA positive rather than neurons, astrocytes, or oligodendrocytes [42], [43]. These results have potential implications for the origin of the stroma in brain tumors. It is possible that the stroma in human gliomas is generated from neural stem/progenitor cells that are either attracted to or induced to proliferate by the tumors, and the cell type they become or whether they differentiate at all will be ultimately influenced by the tumor cells.

In conclusion, stromal cell populations with stem cell-like features can be isolated from human primary tumors and xenografts. While the true cellular origin of these cells is currently unknown, they have a proliferative advantage under certain conditions over tumor cells. This result indicates that care must be taken when preparing cells from glioma tissue *in vitro* and raises the question of the origin of stroma in human gliomas.

## Supporting Information

Figure S1 **Rat stromal cells have a selective growth advantage over EGFR amplified cells in serum monolayer culture. (a)** FISH with an *EGFR*/Chromosome 7 probe in red and green, respectively, and immunohistochemical staining with antibodies against EGFR. In monolayer culture, both EGFR amplification and the chromosome 7 probe are strongly reduced after three months in cultures from P22 xenografts. **(b)** Immunohistochemical staining with antibodies against human-specific and rat-specific nestin. The time course shows that after three months, nestin-expressing rat cells take over at expense of human tumor cells in serum monolayer culture from P22 xenografts. Nestin-expressing human cells are predominant in serum spheroid cultures. Scale bars 50 µm.
(TIF)

Figure S2 **Rat stromal cells have a selective growth advantage over EGFR amplified cells in serum monolayer culture.** Quantification of EGFR, human and rat nestin expressing cells from P22 cultures (3 months) in three random high power (400×) microscopic view fields (HPF) in each group. Values represent mean ± s.d.
(TIF)

Figure S3 **Tumor cells with strongly reduced EGFR expression derived from P22 xenografts have a growth advantage in serum free cultures. (a)** FISH with an *EGFR*/Chromosome 7 probe in red and green, respectively, and immunohistochemical staining with antibodies against EGFR. In P22 cultures both EGFR amplification and the chromosome 7 probe are preserved after two months in culture, but EGFR expression is strongly reduced compared to 1-month-old cultures. Quantification of EGFR expressing cells in three random high power (400×) microscopic view fields (HPF) in each group. Values represent mean ± s.d. **(b)** Immunohistochemical staining with antibodies against human-specific and rat-specific nestin. In P22 cultures human-nestin positive tumor cells have a growth advantage over rat cells. Scale bars 50 µm. Quantification of human and rat nestin expressing cells in three random high power (400×) microscopic view fields (HPF) in each group. Asterix indicates 0%. Values represent mean ± s.d.
(TIF)

Figure S4 **Cultures of an EGFR non-amplified GBM.** Cultures derived from P3 xenografts show polysomy/gain of chromosome 7, but no EGFR amplification. Immunohistochemistry for EGFR is negative. Scale bars 50 µm.
(TIF)

Figure S5
**EGFR non-amplified tumor cells have a growth advantage over rat cells under all growth conditions.** Immunohistochemical staining with antibodies against human-specific and rat-specific nestin. Cultures derived from P3 xenografts show many strongly human-nestin positive and only few rat-nestin positive cells under all conditions. Scale bars 50 .(TIF)

Figure S6 **EGFR non-amplified tumor cells have a growth advantage over rat cells under all growth conditions.** Quantification of human and rat nestin expressing cells from P3 cultures (3 months) in three random high power (400×) microscopic view fields (HPF) in each group. Asterix indicates 0%. Values represent mean ± s.d.
(TIF)

Figure S7 **Stromal cells derived from GBM cultures are non-tumorigenic.** Cells from the different culture conditions derived from P8 xenografts were implanted into the brain of nude rats. T2- weighted MRIs and H&E sections show invasive tumors derived from serum spheroid cultures, while cells derived from monolayer and NBM cultures are non-tumorigenic. Scale bars 50 µm.
(TIF)

Figure S8
**Characterization of rat stromal cells** ***in vitro*** **. (a)** Cells from P22 monolayer cultures were stained with antibodies against GFAP, beta-tubulin III, SOX2 and SMA. The cells are strongly positive for SMA indicating rat stromal cells. The GFAP, beta-tubulin III, and SOX2 positive cells are most likely human tumor cells as there was not a complete selection towards rat stromal cells in P22 monolayer cultures. Scale bars 50 . **(b)** Quantification of GFAP, beta-tubulin, SOX2 and SMA expressing cells from P22 monolayer cultures in three random high power (400×) microscopic view fields (HPF) in each group. Values represent mean ± s.d.(TIF)

Figure S9
**Stromal cells derived from oligodendroglioma cultures are non-tumorigenic.** Cells derived from oligodendroglioma monolayer cultures were implanted into the brain of NOD/SCID mice. H&E section from a NOD/SCID mouse brain shows the injection site devoid of tumor cells. Scale bar 50 .(TIF)

Figure S10
**Characterization of stromal cells derived from oligodendroglioma. (a)** Control PaSMC promoted capillary network formation of co-cultured endothelial cells transduced with GFP for visualization, whereas derived monolayer cultures from oligodendrogliomas did not. **(b)** Monolayer cells from oligodendroglioma were cultured in control medium (DMEM with 10% serum), and osteogenic and adipogenic media for 21 days. Cultures were fixed and treated with alizarin red and oil red to identify bone and fat, respectively. Scale bars 50 .(TIF)

Table S1 **Clinical and genomic information from primary GBM patient biopsies.**
(DOCX)

Table S2 **Derived serum monolayer cell populations from the oligodendroglioma and oligoastrocytoma cases analyzed in the study.**
(DOCX)
