# Supplementary figures and images for: Tumor versus Stromal Cells in Culture—Survival of the Fittest?
Source: PLoS One. 2013 Dec 2;8(12):e81183. doi: 10.1371/journal.pone.0081183 (PMC3857854; doi:10.1371/journal.pone.0081183)

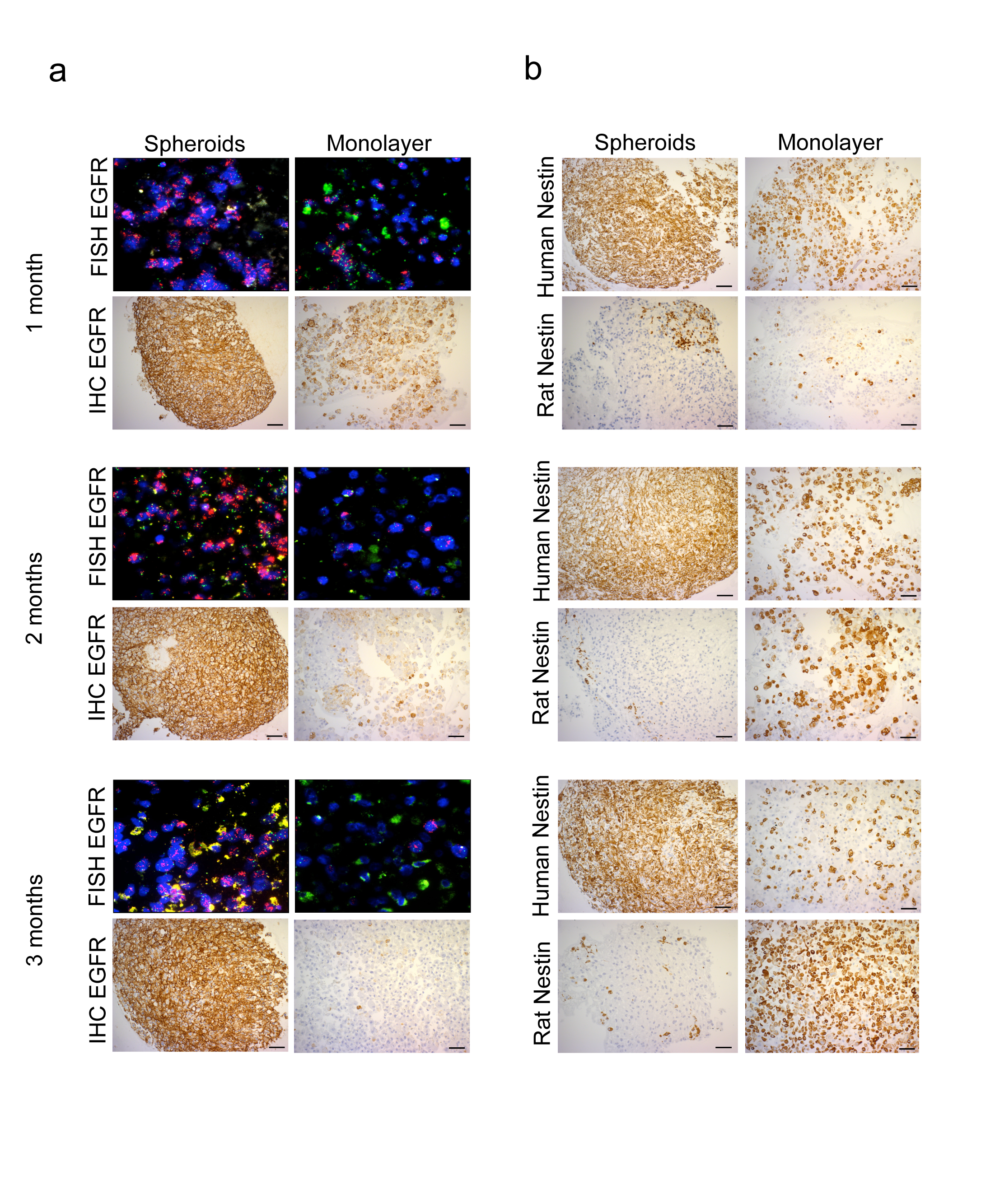

Supplement: Figure S1 — Rat stromal cells have a selective growth advantage over EGFR amplified cells in serum monolayer culture. (a) FISH with an EGFR/Chromosome 7 probe in red and green, respectively, and immunohistochemical staining with antibodies against EGFR. In monolayer culture, both EGFR amplification and the chromosome 7 probe are strongly reduced after three months in cultures from P22 xenografts. (b) Immunohistochemical staining with antibodies against human-specific and rat-specific nestin. The time course shows that after three months, nestin-expressing rat cells take over at expense of human tumor cells in serum monolayer culture from P22 xenografts. Nestin-expressing human cells are predominant in serum spheroid cultures. Scale bars 50 µm. (TIF) [file pone.0081183.s001.tif]

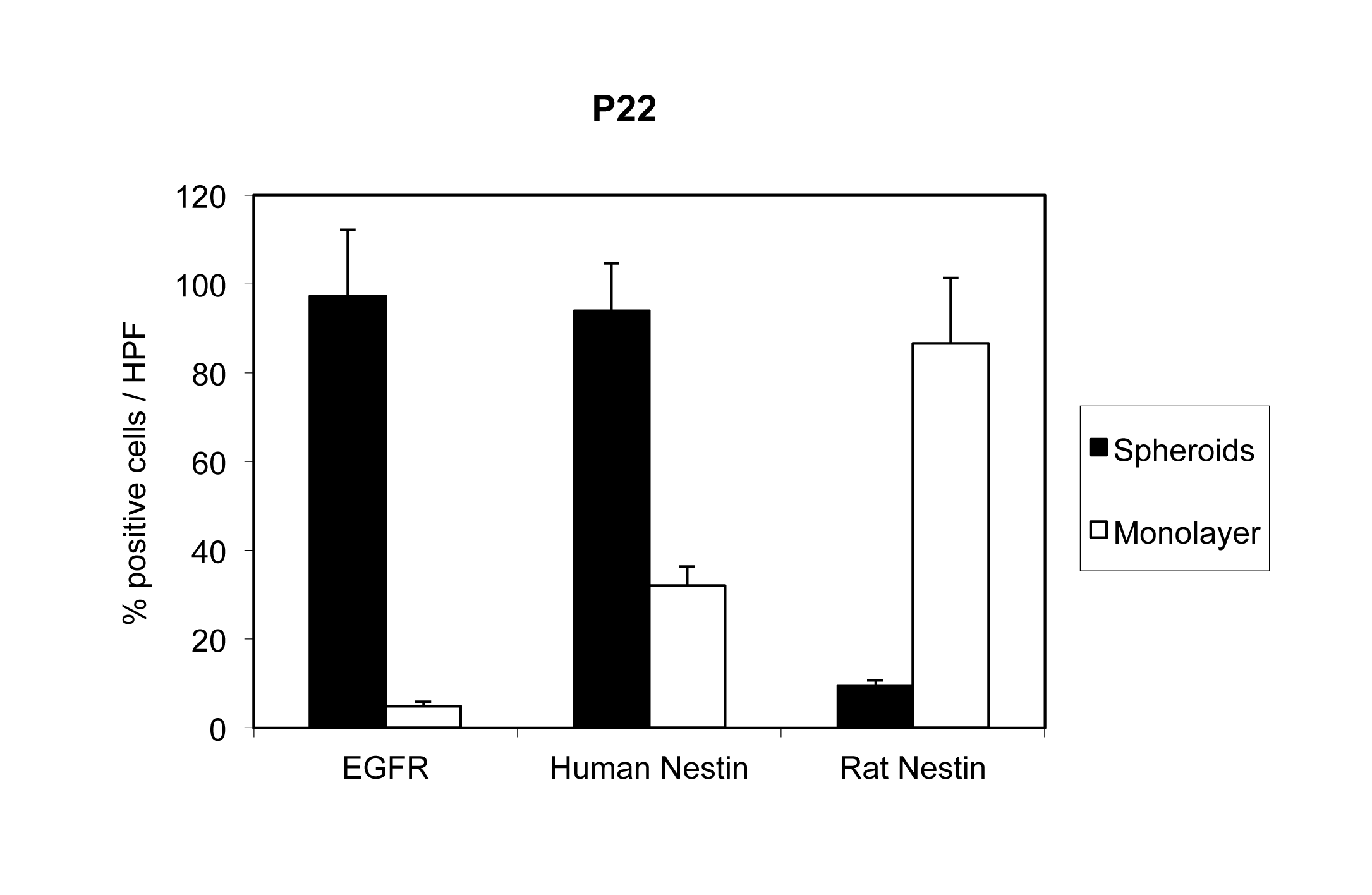

Supplement: Figure S2 — Rat stromal cells have a selective growth advantage over EGFR amplified cells in serum monolayer culture. Quantification of EGFR, human and rat nestin expressing cells from P22 cultures (3 months) in three random high power (400×) microscopic view fields (HPF) in each group. Values represent mean ± s.d. (TIF) [file pone.0081183.s002.tif]

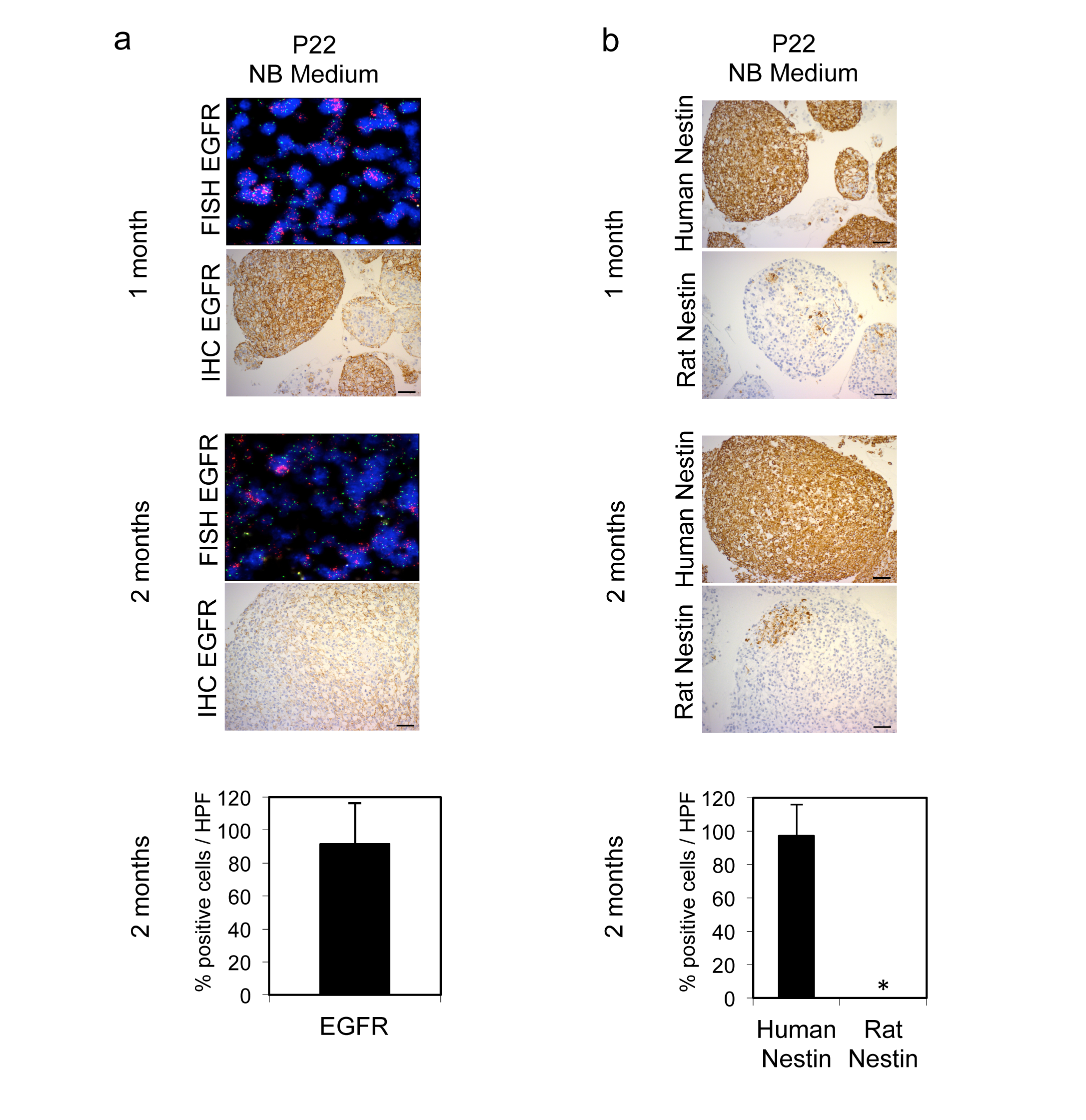

Supplement: Figure S3 — Tumor cells with strongly reduced EGFR expression derived from P22 xenografts have a growth advantage in serum free cultures. (a) FISH with an EGFR/Chromosome 7 probe in red and green, respectively, and immunohistochemical staining with antibodies against EGFR. In P22 cultures both EGFR amplification and the chromosome 7 probe are preserved after two months in culture, but EGFR expression is strongly reduced compared to 1-month-old cultures. Quantification of EGFR expressing cells in three random high power (400×) microscopic view fields (HPF) in each group. Values represent mean ± s.d. (b) Immunohistochemical staining with antibodies against human-specific and rat-specific nestin. In P22 cultures human-nestin positive tumor cells have a growth advantage over rat cells. Scale bars 50 µm. Quantification of human and rat nestin expressing cells in three random high power (400×) microscopic view fields (HPF) in each group. Asterix indicates 0%. Values represent mean ± s.d. (TIF) [file pone.0081183.s003.tif]

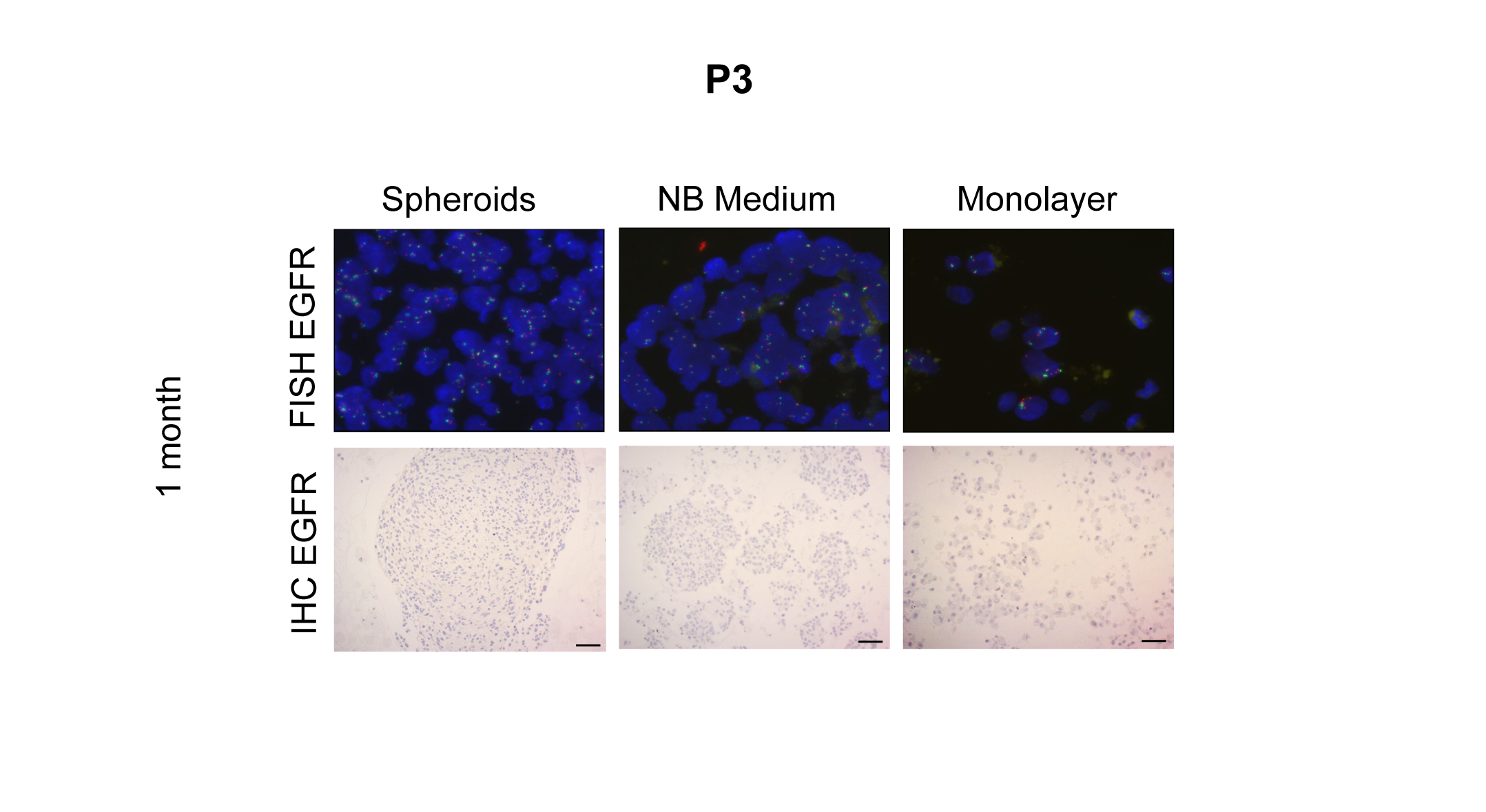

Supplement: Figure S4 — Cultures of an EGFR non-amplified GBM. Cultures derived from P3 xenografts show polysomy/gain of chromosome 7, but no EGFR amplification. Immunohistochemistry for EGFR is negative. Scale bars 50 µm. (TIF) [file pone.0081183.s004.tif]

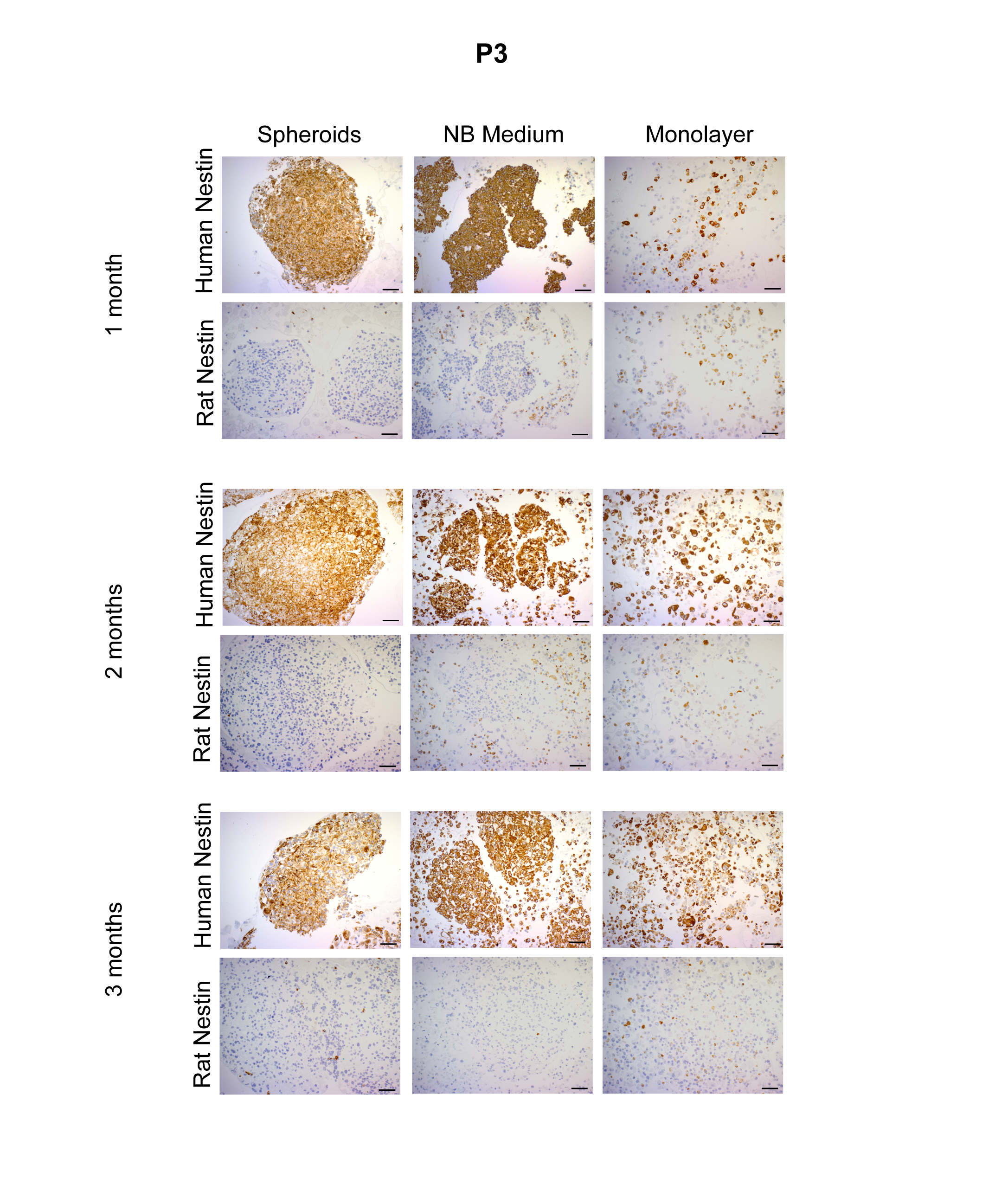

Supplement: Figure S5 — EGFR non-amplified tumor cells have a growth advantage over rat cells under all growth conditions. Immunohistochemical staining with antibodies against human-specific and rat-specific nestin. Cultures derived from P3 xenografts show many strongly human-nestin positive and only few rat-nestin positive cells under all conditions. Scale bars 50 . (TIF) [file pone.0081183.s005.tif]

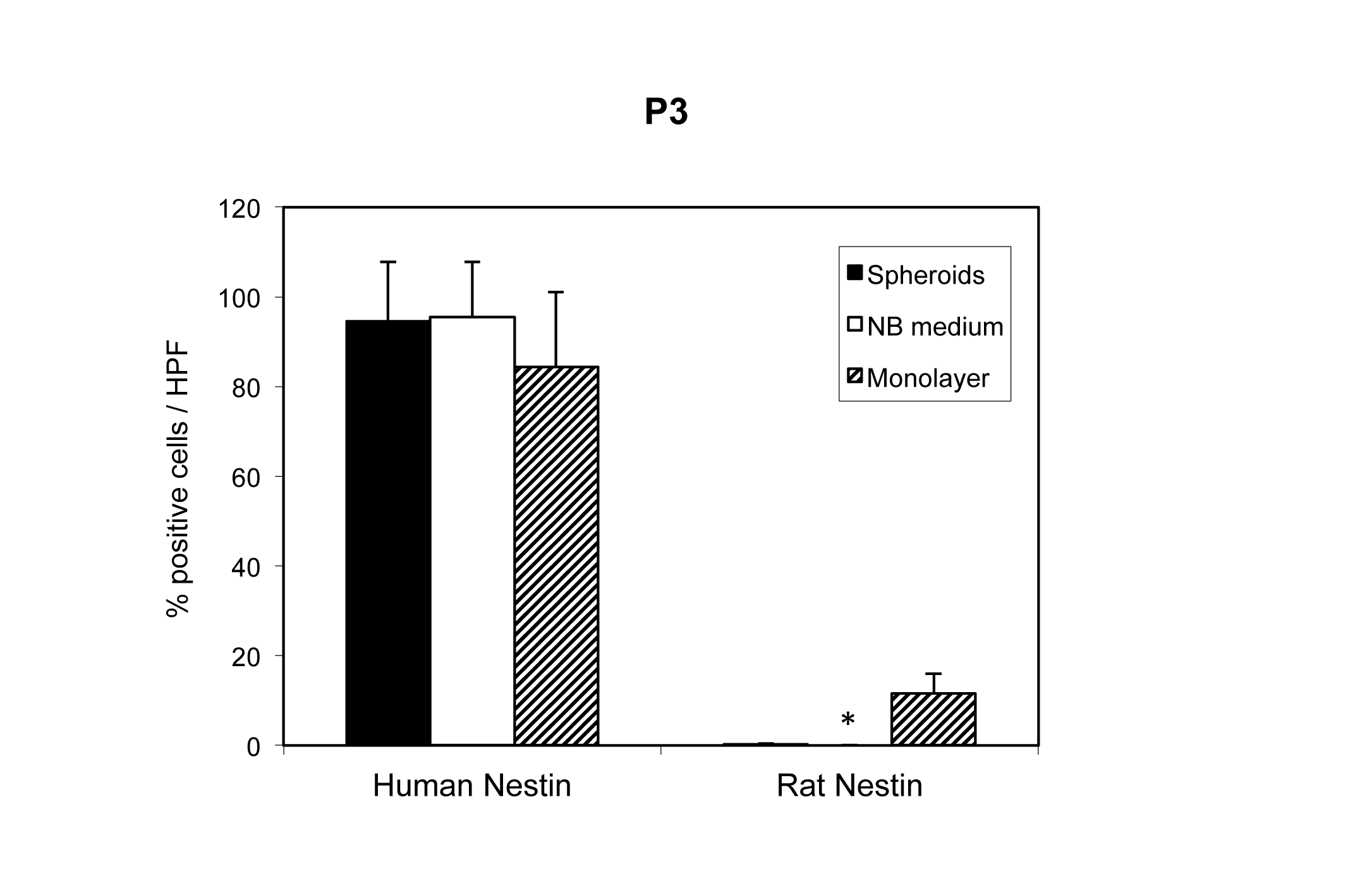

Supplement: Figure S6 — EGFR non-amplified tumor cells have a growth advantage over rat cells under all growth conditions. Quantification of human and rat nestin expressing cells from P3 cultures (3 months) in three random high power (400×) microscopic view fields (HPF) in each group. Asterix indicates 0%. Values represent mean ± s.d. (TIF) [file pone.0081183.s006.tif]

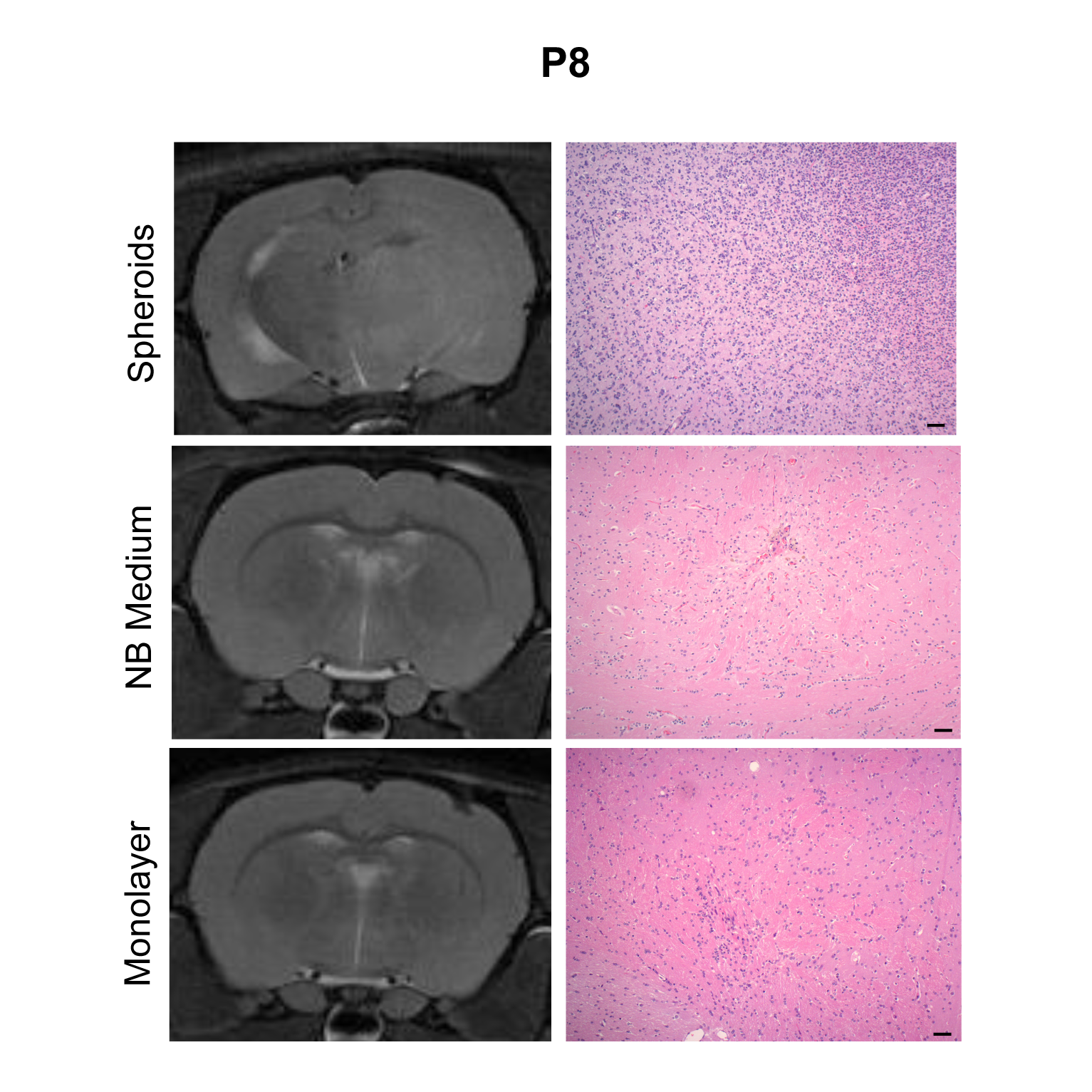

Supplement: Figure S7 — Stromal cells derived from GBM cultures are non-tumorigenic. Cells from the different culture conditions derived from P8 xenografts were implanted into the brain of nude rats. T2- weighted MRIs and H&E sections show invasive tumors derived from serum spheroid cultures, while cells derived from monolayer and NBM cultures are non-tumorigenic. Scale bars 50 µm. (TIF) [file pone.0081183.s007.tif]

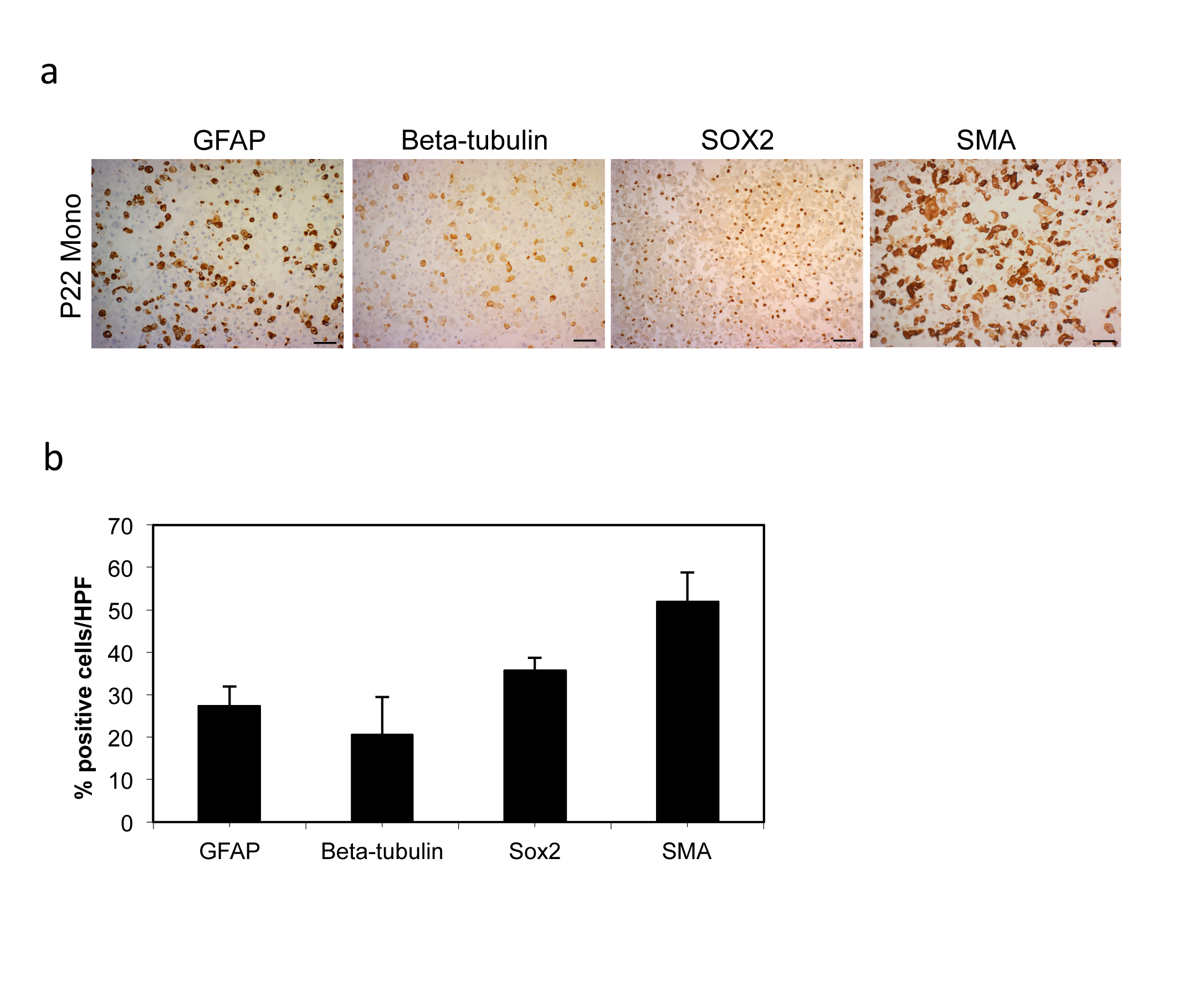

Supplement: Figure S8 — Characterization of rat stromal cells in vitro . (a) Cells from P22 monolayer cultures were stained with antibodies against GFAP, beta-tubulin III, SOX2 and SMA. The cells are strongly positive for SMA indicating rat stromal cells. The GFAP, beta-tubulin III, and SOX2 positive cells are most likely human tumor cells as there was not a complete selection towards rat stromal cells in P22 monolayer cultures. Scale bars 50 . (b) Quantification of GFAP, beta-tubulin, SOX2 and SMA expressing cells from P22 monolayer cultures in three random high power (400×) microscopic view fields (HPF) in each group. Values represent mean ± s.d. (TIF) [file pone.0081183.s008.tif]

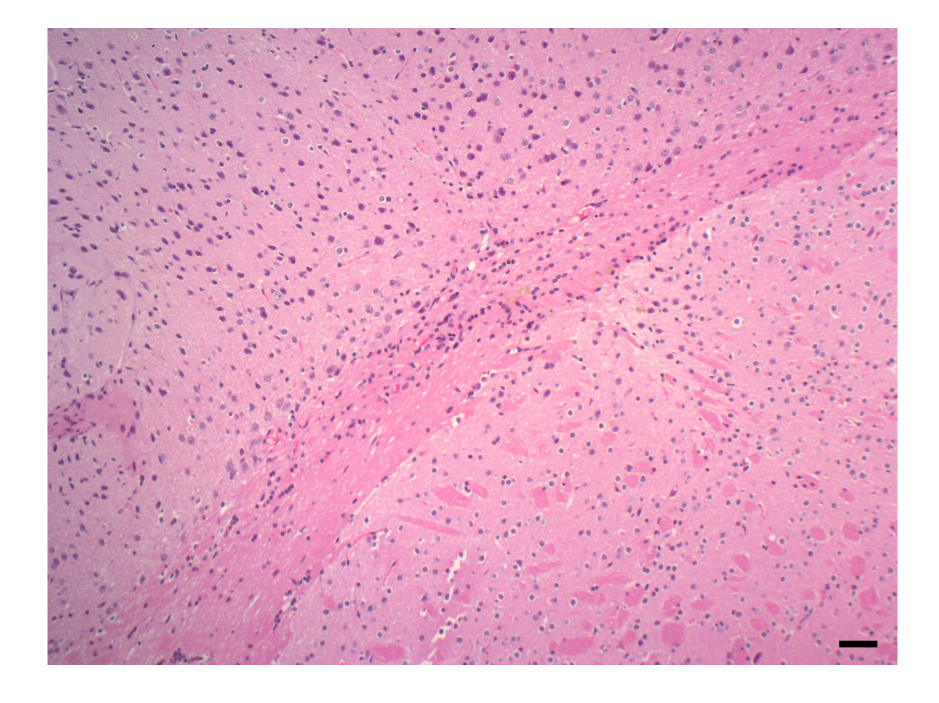

Supplement: Figure S9 — Stromal cells derived from oligodendroglioma cultures are non-tumorigenic. Cells derived from oligodendroglioma monolayer cultures were implanted into the brain of NOD/SCID mice. H&E section from a NOD/SCID mouse brain shows the injection site devoid of tumor cells. Scale bar 50 . (TIF) [file pone.0081183.s009.tif]

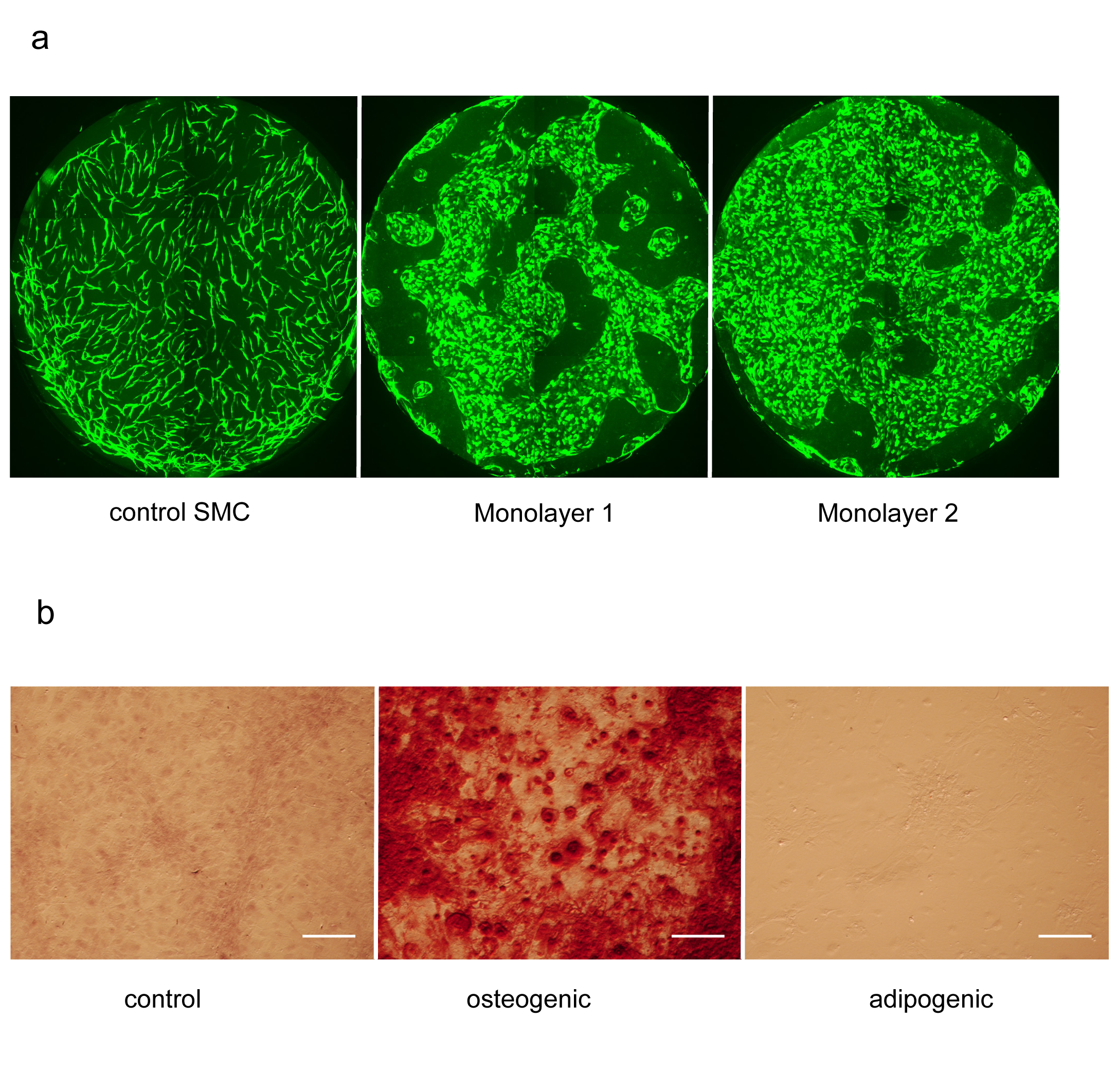

Supplement: Figure S10 — Characterization of stromal cells derived from oligodendroglioma. (a) Control PaSMC promoted capillary network formation of co-cultured endothelial cells transduced with GFP for visualization, whereas derived monolayer cultures from oligodendrogliomas did not. (b) Monolayer cells from oligodendroglioma were cultured in control medium (DMEM with 10% serum), and osteogenic and adipogenic media for 21 days. Cultures were fixed and treated with alizarin red and oil red to identify bone and fat, respectively. Scale bars 50 . (TIF) [file pone.0081183.s010.tif]
