# Supplementary material for: Tumor versus Stromal Cells in Culture—Survival of the Fittest?
Source: PLoS One. 2013 Dec 2;8(12):e81183. doi: 10.1371/journal.pone.0081183 (PMC3857854; doi:10.1371/journal.pone.0081183)
Supplement: Table S1 — Clinical and genomic information from primary GBM patient biopsies. (DOCX) [file pone.0081183.s011.docx]

**Table S1**

**Clinical and genomic information from primary GBM patient biopsies.**

| Patients | age | sex | Chromosomal losses | Chromosomal  gains | *EGFR* amplification | Molecular subtype |
| --- | --- | --- | --- | --- | --- | --- |
| P3 | 64 | male | 9, 10 | 7, 21 | - | Mesenchymal |
| P6 | 80 | female | 5p, 8q, 9p | 3q, 7 | + | Neural |
| P8 | 63 | female | 6q, 9p, 10, 13q, 14p, 18q | 7, 8q | + | Proneural |
| P22 | 69 | male | * | * | + | N/A** |

* In the patient only the EGFR amplicon is detected, however the Xenograft has the typical GBM features (+7, +9q, +19, +20, -10), which is likely due to the fact that the tumor content in the patient sample was very low and did not allow to detect the aberrations due to admixture of normal tissue.

**only data available for Xenograft which is ‘classical subtype‘
