# Supplementary material for: Tumor versus Stromal Cells in Culture—Survival of the Fittest?
Source: PLoS One. 2013 Dec 2;8(12):e81183. doi: 10.1371/journal.pone.0081183 (PMC3857854; doi:10.1371/journal.pone.0081183)
Supplement: Table S2 — Derived serum monolayer cell populations from the oligodendroglioma and oligoastrocytoma cases analyzed in the study. (DOCX) [file pone.0081183.s012.docx]

Table S2. Derived serum monolayer cell populations from the oligodendroglioma and oligoastrocytoma cases analyzed in the study. Biopsy number, diagnosis, genetic test performed of the primary tumor and monolayer cells are indicated.

| Biopsy nr. | diagnosis | IDH1/2 primary tumor | IDH1/2  monolayer | aCGH  monolayer |
| --- | --- | --- | --- | --- |
| 1 | AOA | R132H | normal | nd |
| 2 | O | R132H | normal | nd |
| 3 | O | R132H | normal | normal |
| 4 | O | normal | normal | normal |
| 5 | O | R132H | normal | nd |

AOA: Anaplastic Oligoastrocytoma

O: Oligodendroglioma

nd: not determined
